# Supplementary material for: Short‐Range Order and Li x TM4−x Probability Maps for Disordered Rocksalt Cathodes
Source: Small. 2026 Mar 11;22(22):e14811. doi: 10.1002/smll.202514811 (PMC13089105; doi:10.1002/smll.202514811)
Supplement: Supplementary file 1 — Supporting File: smll72858‐sup‐0001‐SuppMat.pdf. [file SMLL-22-e14811-s001.pdf]

Supporting Information:

Short-Range Order and Li_xTM_{4-x} Probability Maps for Disordered Rocksalt Cathodes

*Tzu-chen Liu   Steven B. Torrisi   Chris Wolverton**

Tzu-chen Liu, Chris Wolverton

Department of Materials Science and Engineering, Northwestern University, Evanston, IL 60208, USA

Email Address: c-wolverton@northwestern.edu

Steven B. Torrisi

Energy & Materials Division, Toyota Research Institute, Los Altos, CA 94022, USA

Derivation of Configuration Matrix

The configuration matrix (C-matrix) shown in Equation 4 can be derived by first writing out the contribution of each tetrahedron configuration to each subcluster correlation:

$$
\begin{pmatrix}
\langle\Gamma_{0000}\rangle \\ \hline
\langle\Gamma_{1000}\rangle \\
\langle\Gamma_{0100}\rangle \\
\langle\Gamma_{0010}\rangle \\
\langle\Gamma_{0001}\rangle \\ \hline
\langle\Gamma_{1100}\rangle \\
\langle\Gamma_{1010}\rangle \\
\langle\Gamma_{1001}\rangle \\
\langle\Gamma_{0110}\rangle \\
\langle\Gamma_{0101}\rangle \\
\langle\Gamma_{0011}\rangle \\ \hline
\langle\Gamma_{1110}\rangle \\
\langle\Gamma_{1101}\rangle \\
\langle\Gamma_{1011}\rangle \\
\langle\Gamma_{0111}\rangle \\ \hline
\langle\Gamma_{1111}\rangle
\end{pmatrix}
=
\left(\begin{array}{c|cccc|cccccc|cccc|c}
+ & + & + & + & + & + & + & + & + & + & + & + & + & + & + & + \\ \hline
+ & - & + & + & + & - & - & - & + & + & + & - & - & - & + & - \\
+ & + & - & + & + & - & + & + & - & - & + & - & - & + & - & - \\
+ & + & + & - & + & + & - & + & - & + & - & - & + & - & - & - \\
+ & + & + & + & - & + & + & - & + & - & - & + & - & - & - & - \\ \hline
+ & - & - & + & + & + & - & - & - & - & + & + & + & - & - & + \\
+ & - & + & - & + & - & + & - & - & + & - & + & - & + & - & + \\
+ & - & + & + & - & - & - & + & + & - & - & - & + & + & - & + \\
+ & + & - & - & + & - & - & + & + & - & - & + & - & - & + & + \\
+ & + & - & + & - & - & + & - & - & + & - & - & + & - & + & + \\
+ & + & + & - & - & + & - & - & - & - & + & - & - & + & + & + \\ \hline
+ & - & - & - & + & + & + & - & + & - & - & - & + & + & + & - \\
+ & - & - & + & - & + & - & + & - & + & - & + & - & + & + & - \\
+ & - & + & - & - & - & + & + & - & - & + & + & + & - & + & - \\
+ & + & - & - & - & - & - & - & + & + & + & + & + & + & - & - \\ \hline
+ & - & - & - & - & + & + & + & + & + & + & - & - & - & - & +
\end{array}\right)
\begin{pmatrix}
P(++++) \\ \hline
P(-+++) \\
P(+-++) \\
P(++-+) \\
P(+++-) \\ \hline
P(--++) \\
P(-+-+) \\
P(-++-) \\
P(+--+) \\
P(+-+-) \\
P(++--) \\ \hline
P(---+) \\
P(--+-) \\
P(-+--) \\
P(+---) \\ \hline
P(----)
\end{pmatrix}. \tag{9}
$$

Here, "+" and "-" denote +1 and -1, respectively. The left-hand side column vector lists each subcluster correlation $\langle\Gamma_{\alpha_1\alpha_2\alpha_3\alpha_4}\rangle$, where α_i is 1 if site i ($i = 1, \ldots, 4$) in the tetrahedron belongs to the cluster α and 0 otherwise. The right-hand side column vector lists probabilities $\mathrm{P}(\sigma_1\sigma_2\sigma_3\sigma_4)$ for all 16 possible occupation combinations on four sites in an tetrahedron, where σ_i is the occupation variable introduced in Equation 2. The matrix in the middle records the cluster correlation values (i.e. the products of occupation variables) for each tetrahedron configuration. The probability-weighted sum of these subcluster correlations over all tetrahedral configurations provides an alternative way to compute the overall averaged values for the entire supercell configuration. Both the cluster indices α_i and the occupation variables σ_1 follow the naming convention of ATAT [49, 59].

To reach Equation 4, we invert the relationship so that the probability column vector appears on the left-hand side. Since the central matrix is orthogonal and symmetric (orthonormal when scaled by 1/4), its inverse is simply itself multiplied by 1/16, which is the coefficient appearing in Equation 4. The final step is to group all equivalent subcluster correlations and tetrahedron probabilities into the reduced column vectors shown in Equation 4.

Summing all of the +1 and -1 in each block (separated by the auxiliary lines) yields the coefficients of the reduced coefficient matrix, which is the final C-matrix used in this work.

Validation of the Short-Range Cluster Approximation

To validate the effectiveness of short-range cluster approximation in capturing cluster energetics and targeted ordering parameters, we compare the standard CE of $LiTMO_2$ (Figure 3) with the fitting the simplified CE that includes only short-range clusters, namely the first two pair clusters and the first four-body cluster, in Table 3. We found that fitted ECIs remained similar between two fittings, suggesting that the approximation retains the correct energetic projections for the energetically dominant short-range clusters. Excluding long-range clusters as additional fitting parameters worsens the CV score, increasing it slightly in the Cu system and by up to a factor of three in the Cr system; on average, the CV score is approximately twice as high across all systems with the short-range approximation, revealing limitations in accurately fitting many small ordered structures.

We then select TM = Cu and Cr systems for further validation of the key ordering parameters, $\alpha_{2,1}$ and Li_4 probability in the disordered states at $T/T_c = 1.1$, to evaluate how the poorer fit affects ordering parameters. MC simulations were performed with a simulation cell of 32^3 sites, with 10,000 MC flips per site for both equilibration and averaging. For Cu, $\alpha_{2,1}$ values are -0.058 and -0.063, and Li_4 probabilities are 0.043 and 0.039 for the standard and simplified CE, respectively. The good performance is not surprising given the similar quality of both fittings. Nevertheless, for Cr, $\alpha_{2,1}$ values are -0.079 and -0.083, and Li_4 probabilities are 0.037 and 0.031 for the standard and simplified CE, respectively. Predictions of high-temperature short-range ordering parameters from the simplified CE remain sufficiently precise, particularly for the primary purpose of comparing with the random-limit Li_4 probability (0.0625). The small discrepancy of approximately 0.005 (less than 10% of the random limit) in Li_4 probabilities can be primarily attributed to the three-body ECI and the correlation $\langle \Gamma_{3,1} \rangle$ (with a coefficient of -4/16 in Equation 5), which is included in the standard CE but omitted from our simplified CE. Analyzing general ordering behavior in the disordered state, rather than constructing a precise Hamiltonian for each $LiTMO_2$, is the primary focus of this study, and the results above confirm that our approximations are effective for this purpose.

Table 3: Comparison between the standard and simplified CE fittings for $LiTMO_2$ (TM = Cr, Cu, Fe, Mn, Ni, and Ti).

	**Cr**	**Cu**	**Fe**	**Mn**	**Ni**	**Ti**
Number of structures	**115**	**97**	**111**	**113**	**110**	**112**
**Standard Cluster Expansion Fitting**						
J_0	-0.026	0.000	0.000	-0.016	-0.011	-0.005
$J_{2,1}$	0.136	0.048	0.085	0.092	0.037	0.098
$J_{2,2}$	0.127	0.056	0.082	0.056	0.049	0.052
$J_{4,1}$	0.000	0.000	-0.003	0.000	0.000	0.000
10-fold CV (meV/site)	18	25	15	26	8	21
**Fitting Using Only Short-range Clusters**						
J_0	-0.012	-0.001	0.007	-0.003	-0.002	0.005
$J_{2,1}$	0.137	0.048	0.077	0.093	0.037	0.098
$J_{2,2}$	0.116	0.055	0.069	0.048	0.046	0.045
$J_{4,1}$	0.009	0.002	-0.002	0.003	0.002	-0.003
10-fold CV (meV/site)	54	29	37	46	19	60

Assessing Projected ECIs as Sufficient Proxies for the ECI Distribution Estimation of $LiTMO_2$

Figure 4 presents the estimated distribution of key ECIs for $LiTMO_2$, which, when translated into the θ parameterization in Figure 9, explains the predominately observed Li_4 probabilities below the random limit for most $LiTMO_2$ compositions. This section provides evidence that such projection based on only four ordering energies (Equation 8), rather than the hundreds of training-structure energies used in the standard CE, can still yield a reasonable estimate of θ that governs the ordering parameters in MC maps. Figure 11 reveals the positive correlation of projected ECIs and the true $\theta = \tan^{-1}(\frac{J_{2,2}}{J_{2,1}})$ from the standard CE, and all the positive $J_{2,1}$ ($\theta \in (-\pi/2, \pi/2)$) are correctly captured by the projected ECI scheme. Moreover, a deviation of θ/π around 0.1 has negligible impact on understanding the ordering trend across $\theta \in [0, 2\pi)$ in Figure 9. This observation supports the effectiveness of the projected ECI in estimating the parameter space of interest for $LiTMO_2$ at a holistic level across $\theta \in [0, 2\pi)$. The error in the projected ECI scheme serves as a reminder of its approximate

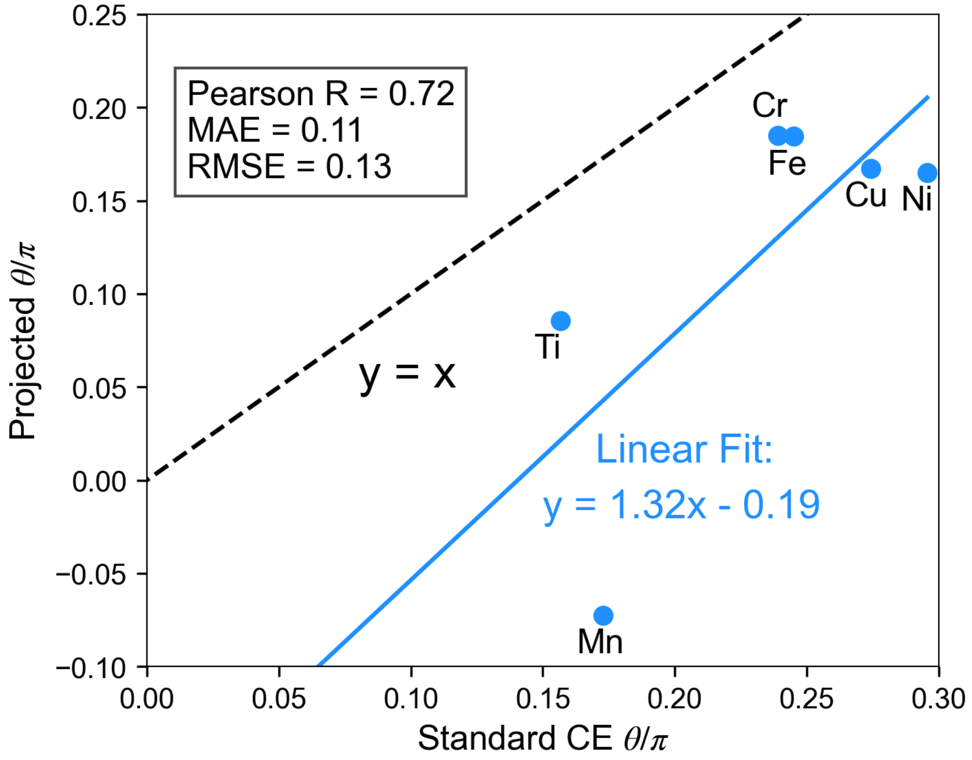

Figure 11: Scattering plot of the θ/π, $\theta = \tan^{-1}(\frac{J_{2,2}}{J_{2,1}}), J_{2,1} > 0$ obtained from the standard CE (Figure 3) and from the projected scheme (Equation 8). Across all chemistries, $J_{2,1}$ is positive in both approaches, which supports one of the central conclusion of this work that positive $J_{2,1}$ ($\theta \in (-\pi/2, \pi/2)$ in Figure 9) is the leading cause of the predominantly observed Li_4 probabilities lower than the random limit. With a positive correlation and errors below the level that could move most systems around or below $\theta/\pi = 0.25$ ($J_{2,2}/J_{2,1} = 1$) beyond $\theta/\pi = 0.5$, the relevant ECI parameter space can be safely defined, while a precise one-to-one correspondence is neither expected nor required in this work.

nature, particularly its consistent underestimation of the normalized $J_{2,2}$ strength relative to that from the standard CE. As noted in the main text, Equation 8 is neither expected nor intended to replace the standard CE fitting method (otherwise, there would be no point in using the much more expensive procedure).

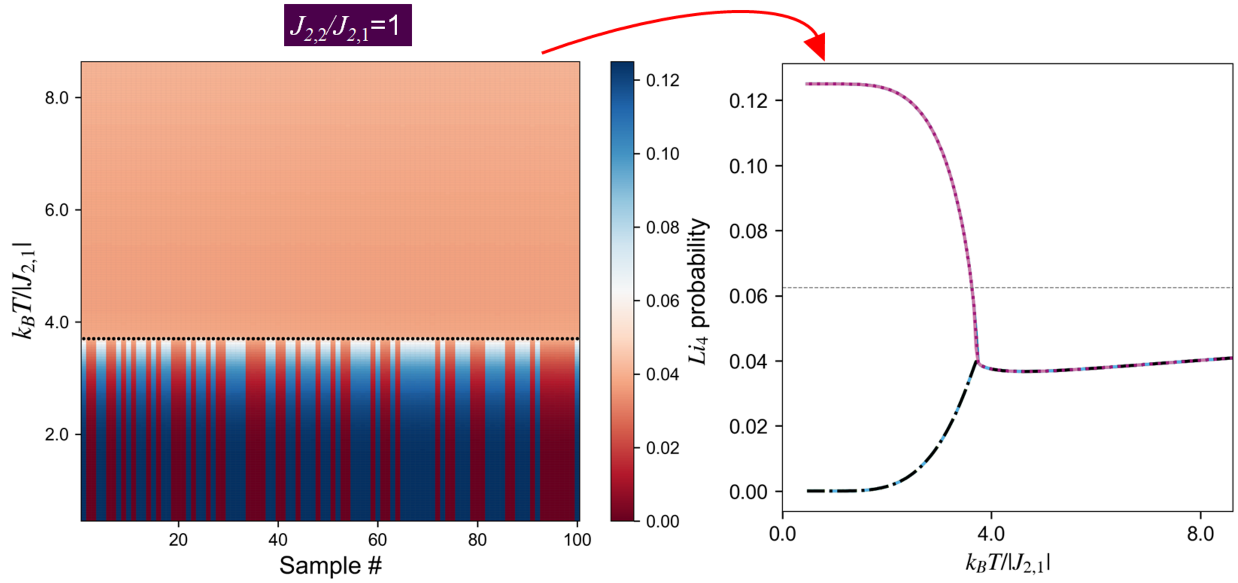

Figure 12: Degeneracy of Layered and Spinel-like LRO when $J_{4,1} = 0$. Simulations with $J_{2,2}/J_{2,1} > 0.5$ below T_c have a 50% probability of equilibrating into either Layered or Spinel-like LRO randomly, as demonstrated in this case of $J_{2,2}/J_{2,1} = 1$ performed 100 times. Regardless of the ground state LRO, all samples have the same high-temperature ordering parameters (including Li_4 probability) as shown in the plot on the right-hand side.
